# (*E*)-2-{1-[(6-Chloro­pyridin-3-yl)meth­yl]imidazolidin-2-yl­idene}-2-cyano-*N*-(2-methylphenyl)acetamide

**DOI:** 10.1107/S1600536811037524

**Published:** 2011-09-30

**Authors:** Jian Wu

**Affiliations:** a Center for Research and Development of Fine Chemicals, Guizhou University, Guiyang, 550025 Guizhou, People’s Republic of China

## Abstract

In the title compound, C_19_H_18_N_5_O, the imidazolidine ring makes dihedral angles of 87.62 (17) and 28.27 (11)° with the pyridine and benzene rings, respectively. An intra­molecular N—H⋯O hydrogen bond is observed between the carbonyl O atom and an imidazolidine H atom. In the crystal, an inter­molecular N—H⋯N hydrogen bond gives rise to a linear chain running along the *b* axis.

## Related literature

For background to neonicotinoids and their biological activity, see: Shao *et al.* (2008 ▶); Nishimura *et al.* (1994 ▶); Mori *et al.* (2002 ▶); Ohno *et al.* (2009 ▶); Tomizawa *et al.* (2000 ▶); Wu *et al.* (2011 ▶).
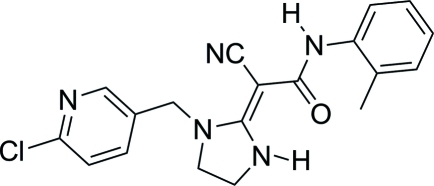


## Experimental

### 

#### Crystal data

C_19_H_18_ClN_5_O
*M* *_r_* = 367.83
Monoclinic, 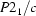

*a* = 16.2019 (18) Å
*b* = 7.6240 (9) Å
*c* = 14.7368 (18) Å
β = 97.007 (3)°
*V* = 1806.7 (4) Å^3^
*Z* = 4
Mo *K*α radiation
μ = 0.23 mm^−1^
*T* = 293 K
0.26 × 0.23 × 0.21 mm

#### Data collection

Bruker APEXII CCD area-detector diffractometer
Absorption correction: multi-scan (*SADABS*; Sheldrick, 1997) ▶ *T*_min_ = 0.943, *T*_max_ = 0.953
19209 measured reflections
3512 independent reflections
2618 reflections with *I* > 2σ(*I*)
*R*_int_ = 0.060

#### Refinement

*R*[*F* ^2^ > 2σ(*F* ^2^)] = 0.049
*wR*(*F* ^2^) = 0.139
*S* = 1.03
3512 reflections
238 parameters
H-atom parameters constrained
Δρ_max_ = 0.22 e Å^−3^
Δρ_min_ = −0.28 e Å^−3^

### 

Data collection: *APEX2* (Bruker, 2002 ▶); cell refinement: *SAINT* (Bruker, 2002 ▶); data reduction: *SAINT*; program(s) used to solve structure: *SHELXS97* (Sheldrick, 2008 ▶); program(s) used to refine structure: *SHELXL97* (Sheldrick, 2008 ▶); molecular graphics: *ORTEP-3 for Windows* (Farrugia, 1997 ▶); software used to prepare material for publication: *WinGX* (Farrugia, 1999 ▶).

## Supplementary Material

Crystal structure: contains datablock(s) global, I. DOI: 10.1107/S1600536811037524/ng5220sup1.cif

Structure factors: contains datablock(s) I. DOI: 10.1107/S1600536811037524/ng5220Isup2.hkl

Supplementary material file. DOI: 10.1107/S1600536811037524/ng5220Isup3.cdx

Supplementary material file. DOI: 10.1107/S1600536811037524/ng5220Isup4.cml

Additional supplementary materials:  crystallographic information; 3D view; checkCIF report

## Figures and Tables

**Table 1:** Hydrogen-bond geometry (Å, °)

*D*—H⋯*A*	*D*—H	H⋯*A*	*D*⋯*A*	*D*—H⋯*A*
N3—H3*A*⋯N4^i^	0.86	2.49	3.044 (3)	123
N3—H3*A*⋯O1	0.86	2.07	2.659 (2)	126

Symmetry code: (i) 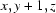.

**Fig. 1.**
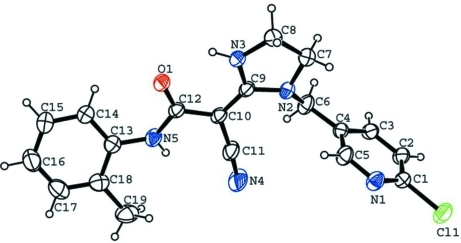
The molecular structure of (I), with displacement ellipsoids drawn at the 30% probability level.

## supplementary crystallographic information

### Comment

Neonicotinoids, an interesting class of insecticide known to act on the central nervous system of insects, are widely used in agriculture due to their broad spectrum activity and low mammalian toxicity. As a part of our ongoing investigation of neonicotinoids analogs, we presented a series of neonicotinoid analogs bearing amide moieties that exhibit good activity against *Nilaparvata lugens* at 100 mg/*L* (Wu *et al.*, 2011). However,the accurate configuration of the active compound in our previous work has not been reported. Herein, we report the crystal structure of the title compound, (*E*)-2-(1-((6-chloropyridin-3-yl)methyl)imidazolidin-2-ylidene)-2- cyano-*N*-(*o*-tolyl)acetamide. It is noteworthy that the crystal of neonicotinoid analog bearing an amide moiety was obtained for the first time.

In the molecule of the title compound (Fig. 1), the imidazoline ring makes dihedral angles of 87.62 (17) ° with pyridine ring and 28.27 (11) ° with benzene ring. An intramolecular N—H···O hydrogen bond is observed between the O atom of carbonyl and imidazoline H atom; The ststructure possesses an intramolecular N3—H3A···O1 hydrogen bond with N3—H3A = 0.86 Å, H3A—O1 = 2.0661 Å, N3—O1 = 2.659 (2) Å, and N—H···O = 125.44 °. In the crystal structure, there are N3—H3A···N4^i^ hydrogen bonds and C—H···π interactions between neighboring molecules, which with the length for bonds N3—H3A, H3A—N4, H3A—N4 were 0.86 Å, 2.4883 Å, 3.044 (3) Å and the angles for N—H···N, C8—H8B···*Cg*(2)^ii^ were 123.03 ° and 113.20 °, respectively; Furthermore, the length for H8B···*Cg*(2)^ii^ and C8···*Cg*(2)^ii^ were 3.1386 Å and 3.632 (3) Å, the angle of C19—H12A···*Cg*(3)^iii^ is 130.96 °; In addition, the length of H12A···*Cg*(3)^iii^ and C19···*Cg*(3)^iii^ were 3.0384 Å and 3.827 (3) Å, respectively [symmetry codes: (i) *x*,-1 + *y*,*z*, (ii) *x*,-1 + *y*,*z*, (iii) *x*,1 - *y*,1 - *z*].

### Experimental

A mixture of 2-cyano-3,3-bis(methylthio)-*N*-(*o*-tolyl)acrylamide (1 mmol) and *N*-((6-chloropyridin-3-yl) methyl) ethane-1,2-diamine (1 mmol) was stirred in refluxing ethanol (10 ml). The progress of the reaction was monitored by TLC. After the completion of the reaction, the mixture was cooled to room temperature, block-shaped crystals were formed, which was filtered off, washed with ethanol and dried in the air.

### Refinement

All H atoms were placed in calculated positions and refined as riding on the parent C atoms with C—H = 0.93–0.97 Å, N—H = 0.86 Å, and *U*_iso_(H) = 1.2 *U*_eq_ (C, N).

### Crystal data

**Table:**

C_19_H_18_ClN_5_O	*F*(000) = 768
*M**_r_* = 367.83	*D*_x_ = 1.352 Mg m^−^^3^
Monoclinic, *P*2_1_/*c*	Mo *K*α radiation, λ = 0.71073 Å
Hall symbol: -P 2ybc	Cell parameters from 19209 reflections
*a* = 16.2019 (18) Å	θ = 1.3–26.0°
*b* = 7.6240 (9) Å	µ = 0.23 mm^−^^1^
*c* = 14.7368 (18) Å	*T* = 293 K
β = 97.007 (3)°	Prism, colourless
*V* = 1806.7 (4) Å^3^	0.26 × 0.23 × 0.21 mm
*Z* = 4	

### Data collection

**Table:**

Bruker APEXII CCD area-detector diffractometer	3512 independent reflections
Radiation source: fine-focus sealed tube	2618 reflections with *I* > 2σ(*I*)
graphite	*R*_int_ = 0.060
φ and ω scans	θ_max_ = 26.0°, θ_min_ = 1.3°
Absorption correction: multi-scan (*SADABS*; Sheldrick, 1997)	*h* = −19→19
*T*_min_ = 0.943, *T*_max_ = 0.953	*k* = −9→9
19209 measured reflections	*l* = −18→17

### Refinement

**Table:**

Refinement on *F*^2^	Primary atom site location: structure-invariant direct methods
Least-squares matrix: full	Secondary atom site location: difference Fourier map
*R*[*F*^2^ > 2σ(*F*^2^)] = 0.049	Hydrogen site location: inferred from neighbouring sites
*wR*(*F*^2^) = 0.139	H-atom parameters constrained
*S* = 1.03	*w* = 1/[σ^2^(*F*_o_^2^) + (0.0717*P*)^2^ + 0.3718*P*] where *P* = (*F*_o_^2^ + 2*F*_c_^2^)/3
3512 reflections	(Δ/σ)_max_ = 0.001
238 parameters	Δρ_max_ = 0.22 e Å^−^^3^
0 restraints	Δρ_min_ = −0.28 e Å^−^^3^

### Special details

**Table:**

Geometry. All e.s.d.'s (except the e.s.d. in the dihedral angle between two l.s. planes) are estimated using the full covariance matrix. The cell e.s.d.'s are taken into account individually in the estimation of e.s.d.'s in distances, angles and torsion angles; correlations between e.s.d.'s in cell parameters are only used when they are defined by crystal symmetry. An approximate (isotropic) treatment of cell e.s.d.'s is used for estimating e.s.d.'s involving l.s. planes.
Refinement. Refinement of *F*^2^ against ALL reflections. The weighted *R*-factor *wR* and goodness of fit *S* are based on *F*^2^, conventional *R*-factors *R* are based on *F*, with *F* set to zero for negative *F*^2^. The threshold expression of *F*^2^ > σ(*F*^2^) is used only for calculating *R*-factors(gt) *etc*. and is not relevant to the choice of reflections for refinement. *R*-factors based on *F*^2^ are statistically about twice as large as those based on *F*, and *R*- factors based on ALL data will be even larger.

### Fractional atomic coordinates and isotropic or equivalent isotropic displacement parameters (Å^2^)

**Table:**

	*x*	*y*	*z*	*U*_iso_*/*U*_eq_	
C1	0.48863 (12)	−0.2075 (2)	−0.41527 (13)	0.0530 (5)	
C2	0.42330 (13)	−0.1992 (3)	−0.48356 (13)	0.0598 (5)	
H2	0.4244	−0.2600	−0.5381	0.072*	
C3	0.35610 (13)	−0.0979 (3)	−0.46850 (13)	0.0552 (5)	
H3	0.3105	−0.0890	−0.5132	0.066*	
C4	0.35663 (11)	−0.0093 (2)	−0.38658 (12)	0.0477 (4)	
C5	0.42631 (13)	−0.0274 (3)	−0.32411 (14)	0.0623 (6)	
H5	0.4278	0.0335	−0.2693	0.075*	
C6	0.28226 (13)	0.0973 (3)	−0.36788 (15)	0.0641 (6)	
H6A	0.2542	0.1407	−0.4254	0.077*	
H6B	0.2437	0.0219	−0.3407	0.077*	
N2	0.30409 (10)	0.2454 (2)	−0.30700 (11)	0.0570 (4)	
C8	0.32548 (15)	0.5482 (3)	−0.28080 (15)	0.0673 (6)	
H8A	0.3771	0.5860	−0.2461	0.081*	
H8B	0.2989	0.6479	−0.3132	0.081*	
C9	0.26236 (11)	0.2939 (2)	−0.23634 (12)	0.0497 (5)	
C10	0.22032 (12)	0.1820 (2)	−0.18154 (13)	0.0506 (5)	
C11	0.23108 (16)	−0.0018 (3)	−0.18395 (18)	0.0735 (6)	
C12	0.17737 (11)	0.2533 (2)	−0.10901 (12)	0.0479 (4)	
H12B	0.1949	−0.1608	0.0393	0.108 (10)*	
H12C	0.1391	−0.2427	0.1011	0.117 (11)*	
H12A	0.1041	−0.2307	−0.0043	0.133 (11)*	
C13	0.10396 (11)	0.1528 (3)	0.02201 (14)	0.0540 (5)	
C14	0.07069 (13)	0.3118 (3)	0.04596 (15)	0.0631 (6)	
H14	0.0718	0.4087	0.0078	0.076*	
C15	0.03602 (16)	0.3252 (4)	0.12656 (17)	0.0790 (7)	
H15	0.0133	0.4313	0.1424	0.095*	
C16	0.03473 (18)	0.1828 (4)	0.18372 (18)	0.0889 (8)	
H16	0.0118	0.1928	0.2384	0.107*	
C17	0.06735 (16)	0.0262 (4)	0.15965 (18)	0.0836 (7)	
H17	0.0658	−0.0694	0.1986	0.100*	
C18	0.10247 (13)	0.0055 (3)	0.07951 (16)	0.0652 (6)	
C19	0.13699 (18)	−0.1683 (3)	0.0544 (2)	0.0883 (8)	
N1	0.49193 (11)	−0.1261 (2)	−0.33656 (12)	0.0644 (5)	
C7	0.33994 (16)	0.4000 (3)	−0.34602 (16)	0.0731 (7)	
H7A	0.3124	0.4240	−0.4069	0.088*	
H7B	0.3989	0.3835	−0.3493	0.088*	
N3	0.27094 (11)	0.4658 (2)	−0.22247 (11)	0.0592 (4)	
H3A	0.2465	0.5223	−0.1828	0.071*	
N4	0.2387 (2)	−0.1517 (3)	−0.1790 (2)	0.1184 (10)	
N5	0.13992 (11)	0.1309 (2)	−0.05957 (12)	0.0611 (5)	
H5A	0.1381	0.0262	−0.0814	0.073*	
O1	0.17555 (9)	0.41125 (17)	−0.09060 (9)	0.0620 (4)	
Cl1	0.57548 (4)	−0.33450 (9)	−0.43072 (4)	0.0805 (2)	

### Atomic displacement parameters (Å^2^)

**Table:**

	*U*^11^	*U*^22^	*U*^33^	*U*^12^	*U*^13^	*U*^23^
C1	0.0587 (11)	0.0458 (10)	0.0560 (11)	0.0075 (8)	0.0131 (9)	−0.0006 (9)
C2	0.0789 (13)	0.0558 (12)	0.0450 (11)	0.0146 (10)	0.0080 (9)	−0.0111 (9)
C3	0.0672 (12)	0.0540 (11)	0.0427 (10)	0.0125 (9)	−0.0005 (8)	−0.0057 (8)
C4	0.0561 (10)	0.0442 (10)	0.0431 (10)	0.0042 (8)	0.0076 (8)	−0.0042 (8)
C5	0.0650 (12)	0.0714 (14)	0.0496 (11)	0.0127 (10)	0.0032 (9)	−0.0184 (10)
C6	0.0623 (12)	0.0744 (14)	0.0548 (12)	0.0141 (10)	0.0040 (9)	−0.0206 (10)
N2	0.0692 (10)	0.0508 (10)	0.0532 (9)	0.0108 (8)	0.0160 (8)	−0.0081 (8)
C8	0.0861 (15)	0.0555 (12)	0.0639 (13)	0.0111 (11)	0.0236 (11)	0.0056 (10)
C9	0.0564 (10)	0.0471 (11)	0.0452 (10)	0.0146 (8)	0.0046 (8)	−0.0048 (8)
C10	0.0572 (11)	0.0408 (10)	0.0540 (11)	0.0068 (8)	0.0072 (8)	−0.0082 (8)
C11	0.0889 (16)	0.0509 (14)	0.0875 (16)	0.0048 (11)	0.0375 (13)	−0.0124 (11)
C12	0.0520 (10)	0.0436 (10)	0.0473 (10)	0.0052 (8)	0.0027 (8)	−0.0048 (8)
C13	0.0453 (10)	0.0568 (12)	0.0600 (12)	−0.0044 (8)	0.0067 (8)	−0.0024 (9)
C14	0.0614 (12)	0.0642 (14)	0.0654 (13)	0.0096 (10)	0.0145 (10)	0.0006 (10)
C15	0.0834 (16)	0.0865 (18)	0.0714 (15)	0.0118 (13)	0.0267 (13)	−0.0074 (13)
C16	0.0936 (19)	0.108 (2)	0.0700 (16)	0.0016 (16)	0.0287 (14)	0.0055 (16)
C17	0.0858 (17)	0.0905 (19)	0.0765 (17)	−0.0106 (14)	0.0182 (14)	0.0223 (14)
C18	0.0568 (11)	0.0588 (13)	0.0791 (15)	−0.0077 (10)	0.0051 (11)	0.0058 (11)
C19	0.0933 (19)	0.0552 (15)	0.117 (2)	−0.0042 (13)	0.0150 (16)	0.0151 (15)
N1	0.0613 (10)	0.0739 (12)	0.0561 (10)	0.0134 (9)	−0.0004 (8)	−0.0134 (9)
C7	0.0949 (17)	0.0642 (14)	0.0654 (14)	0.0189 (12)	0.0303 (12)	0.0051 (11)
N3	0.0820 (11)	0.0422 (9)	0.0569 (10)	0.0107 (8)	0.0229 (8)	−0.0018 (7)
N4	0.178 (3)	0.0454 (13)	0.150 (2)	0.0096 (14)	0.092 (2)	−0.0105 (13)
N5	0.0693 (11)	0.0447 (9)	0.0724 (12)	−0.0027 (8)	0.0210 (9)	−0.0106 (8)
O1	0.0873 (10)	0.0426 (8)	0.0596 (9)	0.0034 (7)	0.0229 (7)	−0.0087 (6)
Cl1	0.0730 (4)	0.0846 (5)	0.0850 (5)	0.0292 (3)	0.0141 (3)	−0.0110 (3)

### Geometric parameters (Å, °)

**Table:**

C1—N1	1.311 (3)	C10—C12	1.450 (3)
C1—C2	1.370 (3)	C11—N4	1.151 (3)
C1—Cl1	1.7458 (19)	C12—O1	1.236 (2)
C2—C3	1.375 (3)	C12—N5	1.370 (3)
C2—H2	0.9300	C13—C14	1.390 (3)
C3—C4	1.383 (3)	C13—N5	1.408 (3)
C3—H3	0.9300	C13—C18	1.408 (3)
C4—C5	1.374 (3)	C14—C15	1.378 (3)
C4—C6	1.506 (3)	C14—H14	0.9300
C5—N1	1.334 (3)	C15—C16	1.376 (4)
C5—H5	0.9300	C15—H15	0.9300
C6—N2	1.459 (3)	C16—C17	1.370 (4)
C6—H6A	0.9700	C16—H16	0.9300
C6—H6B	0.9700	C17—C18	1.381 (4)
N2—C9	1.360 (2)	C17—H17	0.9300
N2—C7	1.462 (3)	C18—C19	1.502 (4)
C8—N3	1.449 (3)	C19—H12B	0.9917
C8—C7	1.520 (3)	C19—H12C	0.8887
C8—H8A	0.9700	C19—H12A	1.0711
C8—H8B	0.9700	C7—H7A	0.9700
C9—N3	1.331 (2)	C7—H7B	0.9700
C9—C10	1.407 (3)	N3—H3A	0.8600
C10—C11	1.413 (3)	N5—H5A	0.8600
			
N1—C1—C2	124.94 (18)	N5—C12—C10	114.85 (16)
N1—C1—Cl1	115.56 (15)	C14—C13—N5	122.25 (19)
C2—C1—Cl1	119.50 (15)	C14—C13—C18	120.5 (2)
C1—C2—C3	117.60 (18)	N5—C13—C18	117.29 (19)
C1—C2—H2	121.2	C15—C14—C13	119.7 (2)
C3—C2—H2	121.2	C15—C14—H14	120.2
C2—C3—C4	119.67 (18)	C13—C14—H14	120.2
C2—C3—H3	120.2	C16—C15—C14	120.5 (2)
C4—C3—H3	120.2	C16—C15—H15	119.8
C5—C4—C3	116.93 (17)	C14—C15—H15	119.8
C5—C4—C6	122.82 (17)	C17—C16—C15	119.6 (2)
C3—C4—C6	120.23 (17)	C17—C16—H16	120.2
N1—C5—C4	124.64 (18)	C15—C16—H16	120.2
N1—C5—H5	117.7	C16—C17—C18	122.2 (2)
C4—C5—H5	117.7	C16—C17—H17	118.9
N2—C6—C4	113.00 (17)	C18—C17—H17	118.9
N2—C6—H6A	109.0	C17—C18—C13	117.6 (2)
C4—C6—H6A	109.0	C17—C18—C19	121.1 (2)
N2—C6—H6B	109.0	C13—C18—C19	121.4 (2)
C4—C6—H6B	109.0	C18—C19—H12B	113.3
H6A—C6—H6B	107.8	C18—C19—H12C	110.6
C9—N2—C6	125.12 (18)	H12B—C19—H12C	105.3
C9—N2—C7	109.92 (16)	C18—C19—H12A	115.3
C6—N2—C7	117.40 (17)	H12B—C19—H12A	103.6
N3—C8—C7	101.83 (18)	H12C—C19—H12A	108.1
N3—C8—H8A	111.4	C1—N1—C5	116.21 (17)
C7—C8—H8A	111.4	N2—C7—C8	104.54 (17)
N3—C8—H8B	111.4	N2—C7—H7A	110.8
C7—C8—H8B	111.4	C8—C7—H7A	110.8
H8A—C8—H8B	109.3	N2—C7—H7B	110.8
N3—C9—N2	109.43 (17)	C8—C7—H7B	110.8
N3—C9—C10	123.91 (16)	H7A—C7—H7B	108.9
N2—C9—C10	126.56 (17)	C9—N3—C8	113.30 (16)
C9—C10—C11	121.14 (18)	C9—N3—H3A	123.3
C9—C10—C12	120.36 (16)	C8—N3—H3A	123.3
C11—C10—C12	117.51 (19)	C12—N5—C13	129.02 (17)
N4—C11—C10	174.7 (3)	C12—N5—H5A	115.5
O1—C12—N5	121.52 (17)	C13—N5—H5A	115.5
O1—C12—C10	123.60 (18)		

### Hydrogen-bond geometry (Å, °)

**Table:**

*D*—H···*A*	*D*—H	H···*A*	*D*···*A*	*D*—H···*A*
N3—H3A···N4^i^	0.86	2.49	3.044 (3)	123.
N3—H3A···O1	0.86	2.07	2.659 (2)	126.

Symmetry codes: (i) *x*, *y*+1, *z*.
